# Multicenter Study of the Hemorrhage Risk after Endoscopic Mucosal Resection Associated with Direct Oral Anticoagulants

**DOI:** 10.1155/2019/5743561

**Published:** 2019-12-18

**Authors:** Ritsu Yasuda, Naohisa Yoshida, Takaaki Murakami, Ryohei Hirose, Ken Inoue, Osamu Dohi, Yuji Naito, Yutaka Inada, Takashi Okuda, Daisuke Hasegawa, Kotaro Okuda, Kiyoshi Ogiso, Yoshikazu Inagaki, Koichi Soga, Akira Tomie, Yoshito Itoh

**Affiliations:** ^1^Department of Molecular Gastroenterology and Hepatology, Kyoto Prefectural University of Medicine, Graduate School of Medical Science, Kyoto, Japan; ^2^Department of Gastroenterology, JCHO Kyoto Kuramaguchi Medical Center, Kyoto, Japan; ^3^Department of Gastroenterology, North Medical Center Kyoto Prefectural University of Medicine, Kyoto, Japan; ^4^Department of Gastroenterology, Fukuchiyama City Hospital, Kyoto, Japan; ^5^Department of Gastroenterology, Ayabe City Hospital, Kyoto, Japan; ^6^Department of Gastroenterology, Kyoto Kujo Hospital, Kyoto, Japan; ^7^Department of Gastroenterology, Osaka General Hospital of West Japan Railway Company, Osaka, Japan; ^8^Department of Gastroenterology, Nishijin Hospital, Kyoto, Japan; ^9^Department of Gastroenterology, Kyoto Yamashiro General Medical Center, Kyoto, Japan

## Abstract

**Backgrounds and Aims:**

Recently, direct oral anticoagulants (DOACs) have become widely used for preventing thromboembolism. However, postoperative hemorrhage (POH) is a major complication associated with endoscopic mucosal resection (EMR) for colorectal lesions. In this multicenter study, we analyzed the incidence of POH after EMR associated with DOACs and explored the associated risk factors.

**Materials and Methods:**

This study was a multicenter retrospective cohort study conducted at 8 Japanese institutions. A total of 2062 cases that underwent EMR for colorectal lesions at these 8 institutions from October 2016 to September 2017 were analyzed. The cases were divided into 4 groups: the DOAC group (63 cases), warfarin group (34 cases), antiplatelet group (185 cases), and no antithrombotics group (1780 cases). In all lesions of the DOAC and warfarin groups, endoscopic clipping was performed after EMR. The rate of POH in the DOAC group, patients' clinical characteristics, the risk factors of POH, and the rate of thromboembolism due to stopping DOACs were compared with other groups.

**Results:**

The rates of POH were 7.9%^∗^ (5/63), 2.9% (1/34), 3.2% (6/185), and 0.6%^∗∗^ (11/1780) in the DOAC, warfarin, antiplatelet, and no antithrombotics groups, respectively (^∗^ vs. ^∗∗^, *p* < 0.001). Regarding risk factors, the tumor size with POH (mm) was significantly bigger than that without POH (16.2 ± 8.3 vs. 7.2 ± 4.9, *p* < 0.001). There were no significant differences in the rates of POH based on the type of DOAC. In addition, no thromboembolisms occurred due to stopping of DOAC treatment.

**Conclusions:**

Patients receiving DOACs had significantly higher rates of POH after EMR than those without antithrombotics.

## 1. Introduction

With the growing elderly population, the number of patients receiving antithrombotics is increasing globally [1]. Similarly, rates of colorectal neoplasia, such as colorectal cancer and colorectal adenoma, are increasing due to the aging of the population [2]. Endoscopic mucosal resection (EMR) for removing colorectal neoplasia is widely performed worldwide [3]. Concurrently, the number of patients receiving antithrombotics is increasing along with the age at which colonoscopic examinations are performed [4]. Physicians should therefore be familiar with performing EMR in patients receiving these drugs.

Direct oral anticoagulants (DOACs) are antithrombotic drugs that have been used to prevent cerebrovascular disease and deep vein thrombosis for about a decade [5]. In 2018, four types of DOACs (dabigatran, rivaroxaban, apixaban, and edoxaban) were approved for use in Japan. Compared with warfarin, DOACs have superior pharmacological properties, including (i) a better and rapid dose response, (ii) less difference in anticoagulant activity between individuals, (iii) no influence by vitamin K intake, and (iv) very few drug interactions. However, the risk of non-procedural-related gastrointestinal (GI) bleeding is higher with some DOACs than with warfarin [6].

Postoperative hemorrhage (POH) is a major complication associated with EMR for colorectal neoplasms, and the risk of POH is reportedly increased in patients receiving antithrombotics compared with those not receiving antithrombotics [7, 8]. Management of antithrombotics, including DOACs, in patients undergoing endoscopic procedures has become an important issue and was described in the guidelines of the American Society for Gastrointestinal Endoscopy (ASGE), the European Society of Gastrointestinal Endoscopy (ESGE), the British Society for Gastroenterology (BSGE), and the Japan Gastroenterological Endoscopy Society (JGES) [9–12]. However, few large-scale studies have evaluated the risk of POH after EMR in patients taking four types of DOACs. In addition, there is a distinct lack of multicenter studies investigating POH in patients undergoing EMR.

In the present study, we analyzed the rate of POH after EMR associated with DOACs and evaluated the associated risk factors.

## 2. Materials and Methods

This study was a multicenter retrospective cohort study conducted at eight Japanese institutions: Kyoto Prefectural University of Medicine, North Medical Center Kyoto Prefectural University of Medicine, Fukuchiyama City Hospital, Ayabe City Hospital, Kyoto Kujo Hospital, Osaka General Hospital of West Japan Railway Company, Nishijin Hospital, and Kyoto Yamashiro General Medical Center. A total of 2062 cases that underwent EMR for colorectal neoplasms at these 8 institutions from October 2016 to September 2017 were analyzed.

**Figure 1:**
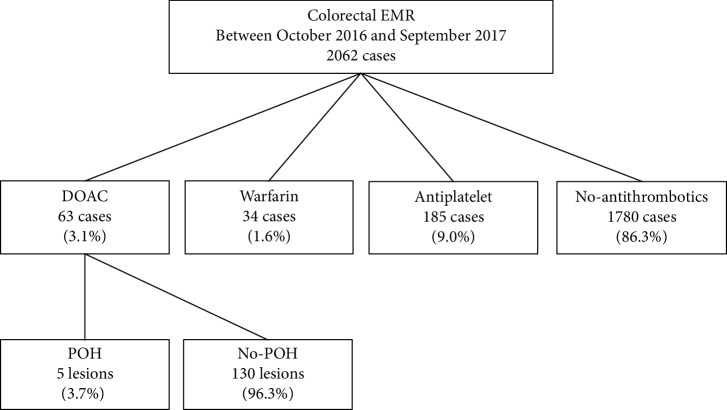
Study flow.

The cases were divided into 4 groups: the DOAC group (63 cases), warfarin group (34 cases), antiplatelet group (185 cases), and no antithrombotics group (1780 cases) (Figure 1). The DOAC and warfarin groups included cases with coprescription of antiplatelets. All lesions in the DOAC and warfarin groups received endoscopic clipping after EMR. In the other two groups, endoscopic clipping was performed according to the operator's decision.

The rate of POH, clinical characteristics, and thromboembolic events were compared among the four groups. In addition, lesions with POH were compared to those without POH in order to analyze the risk factors of POH in DOAC cases. Furthermore, in the DOAC group, the rates of POH were calculated for each specific DOAC.

POH was defined when the hemoglobin level decreased by >2 g/dL or in cases of apparent bleeding or massive melena within 1 month after EMR [13]. A thromboembolic event was defined as the occurrence of acute coronary syndrome, stroke, pulmonary embolism, or deep vein thrombosis from one week before EMR to one month after EMR.

The management of antithrombotics was performed according to the Japan Gastroenterological Endoscopy Society guidelines for gastrointestinal endoscopy for patients taking antithrombotics in 2012 [11]. In detail, DOACs were discontinued one day before EMR, and their readministration was performed in the morning on the day after EMR. Heparin bridging was not regularly performed. Warfarin was replaced with heparin three to five days before EMR, and intravenous infusion of heparin was suspended at least three hours before EMR. Administration of both warfarin and heparin was resumed the day after EMR. Heparin was discontinued when the prothrombin time-international normalized ratio (PT-INR) returned to the therapeutic range. Some patients receiving aspirin monotherapy had their treatment withdrawn for three to five days before EMR, although others did not have it withdrawn due to a high risk of thromboembolism. Thienopyridine derivatives (ticlopidine, clopidogrel, and prasugrel) were withdrawn for five to seven days. Other antiplatelets (cilostazol, ethyl icosapentate, sarpogrelate, beraprost, and limaprost alfadex) were withdrawn for one day. Administration of antiplatelets was resumed the day after EMR.

The polyp locations were divided into three parts: the right-sided colon (from the cecum to the transverse colon), left-sided colon (from the descending colon to the sigmoid colon), and rectum. Morphologically, the polyps were divided into polypoid and nonpolypoid lesions according to the Paris classification [14]. The specimens resected by EMR were placed in formalin as en bloc tissue after resection. The specimens (hematoxylin and eosin staining) were evaluated by authorized pathologists. The histopathological diagnosis was according to Japanese Scosiety of Cancer of the Colon and and Rectum (JSCCR). Especially, sessile serrated adenoma and polyp (SSA/P) was distinguished from hyperplastic polyp according to the JSCCR criteria, as follows: (1) dilatation of ducts, (2) irregularly branched ducts, and (3) horizontally arranged basal ducts (inverted T shape or L shape) [15]. SSA/Ps were diagnosed when at least 10% of the lesions had 2 of these 3 findings. Cases of mild and moderate dysplasia were diagnosed as adenoma, while cases with severe dysplasia and intramucosal cancer were diagnosed as high-grade dysplasia (HGD). A negative margin was defined when a lesion gland and cells were not detected on the definite resected margin.

### 2.1. EMR

The patient's bowels were prepared by the consumption of 1.0 L of highly concentrated polyethylene glycol solution (EA Pharma Co., Tokyo, Japan) or 2.0 L of polyethylene glycol solution the morning before the examination [16]. We used a lower GI endoscope with a single channel. For the injection solution, we used saline or 0.13% HA solution. The 0.13% HA solution was prepared by diluting 0.4% HA solution (Mucoup, Johnson & Johnson, Tokyo, Japan, or Seikagaku Corporation, Tokyo, Japan) with NS [17]. Various snares 15-25 mm in size were used with an automatically controlled high-frequency generator (VIO300D or ICC200, Erbe Elektromedizin Ltd., Tübingen, Germany, or ESG100, Olympus Co., Tokyo, Japan).

### 2.2. Statistical Analyses

Statistical analyses were performed using the Mann–Whitney *U* test and a one-way analysis of variance (ANOVA). Continuous variables, such as the patient age and tumor size, were analyzed using the Mann–Whitney *U* test. Categorical variables, such as the rate of POH and other endpoints, were analyzed using a one-way ANOVA. Statistical analyses were performed using the GraphPad Prism software program (ver. 6.0; GraphPad Software, La Jolla, CA, USA). *p* values less than 0.05 were considered statistically significant.

## 3. Results

In the DOAC, warfarin, antiplatelet, and no antithrombotics groups, the mean ages were 74.2 ± 7.0^∗^, 73.2 ± 6.7^∗^, 72.1 ± 8.8^∗^, and 66.3±11.1^∗∗^ years old (^∗^ vs. ^∗∗^, *p* < 0.001), and the rates of male gender were 66.7% (42/63), 67.6% (23/34), 75.1%^∗^ (139/185), and 61.8%^∗∗^ (1100/1780) (^∗^ vs. ^∗∗^, *p* < 0.001), respectively (Table 1). There were no significant differences in the mean polyp size or rate of right-sided colon among the four groups. In the DOAC group, the proportions of each DOAC (rivaroxaban, apixaban, edoxaban, and dabigatran) were 44.4% (28/63), 25.4% (16/63), 19.0% (12/63), and 11.1% (7/63), respectively. In the antiplatelet group, the rate of single-agent use was 80.0% (148/185), two-drug combination was 18.9% (35/185), and three-drug combination was 0.1% (2/185). Regarding the type of antiplatelet agent, there were 105 cases using aspirin, 39 cases using thienopyridine derivatives, and 63 cases using other antiplatelet agents.

In the DOAC group, lesions with and without POH were compared (Table 2). There were no significant differences in the morphology, tumor location, histology, or coprescription of antiplatelets between the two subgroups. However, a significant difference in the polyp size was observed between lesions with and without POH (16.2 ± 8.3 mm vs. 7.2 ± 4.9 mm, respectively; *p* < 0.001).

**Figure 2:**
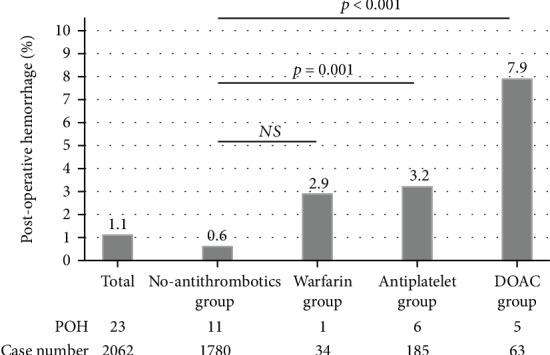
The rates of postoperative hemorrhage after endoscopic mucosal resection in the no antithrombotics, warfarin, antiplatelet, and DOAC groups.

**Figure 3:**
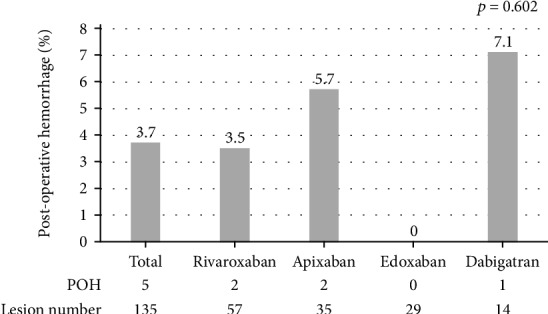
The rates of postoperative hemorrhage after endoscopic mucosal resection per lesion by each DOAC.

The total rate of POH for all cases was 1.1% (23/2062). The rates of POH were 7.9%^∗^ (5/63), 2.9% (1/34), 3.2%^∗∗^ (6/185), and 0.6%^∗∗∗^ (11/1780) in the DOAC, warfarin, antiplatelet, and no antithrombotics groups, respectively (^∗^ vs. ^∗∗∗^, *p* < 0.001, ^∗∗^ vs. ^∗∗∗^, *p* = 0.001) (Figure 2). No thromboembolic events were observed in any group. Regarding the types of DOACs, we analyzed the rates of POH per lesion. The overall rate was 3.7% (5/135, 95% CI: 1.21-8.43) and the rates for each DOAC were 3.5% (2/57, 95% CI: 0.4-12.1), 5.7% (2/35, 95% CI: 0.7-19.1), 0% (0/29, 95% CI: 0-11.9), and 7.1% (1/14, 95% CI: 0.18-33.8) in the rivaroxaban, apixaban, edoxaban, and dabigatran users, respectively (*p* = 0.602) (Figure 3).

**Figure 4:**
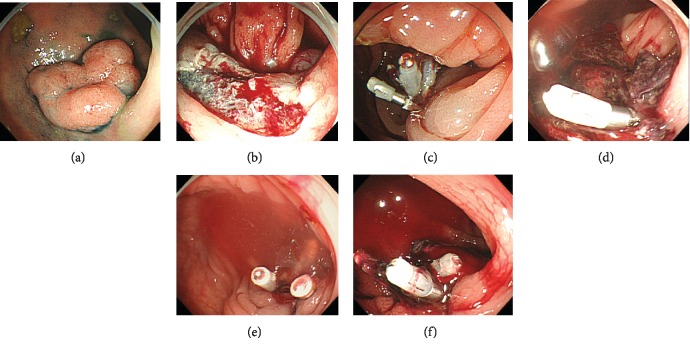
A case treated with DOACs in which postoperative hemorrhage occurred twice after EMR. (a) A 76-year-old man receiving apixaban with no antiplatelets and hemodialysis. A nonpolypoid lesion 25 mm in size on the transverse colon. (b) EMR was performed. (c) Endoscopic clipping was performed in the EMR ulcer. (d) Postoperative hemorrhage occurred two days after EMR. One previous endoscopic clipping remained in the EMR ulcer. (e) Endoscopic clipping was performed again for hemostasis. (f) Postoperative hemorrhage occurred a second time three days after EMR. Endoscopic clipping was performed again, and no recurrent postoperative hemorrhage occurred after this round of hemostasis.

The details of the five patients receiving DOAC with POH are summarized in Table 3. Regarding the tumor size, 3 of them were ≥20 mm. There were no cases with coprescription of antiplatelets. POH occurred 1 to 10 days after EMR, and 2 cases experienced POH twice (Figure 4).

## 4. Discussion

In this multicenter study, the rate of POH after EMR in the DOAC group was 7.9%, which was significantly higher than the no antithrombotics group. A larger tumor size especially ≥20 mm was deemed a risk factor of POH in patients with DOAC.

Singh et al. reported that the rate of POH after polypectomy for colorectal neoplasia was significantly higher in patients taking clopidogrel than patients without it [18]. Pan et al. reported that the rate of POH after therapeutic colonoscopy for colorectal neoplasia in patients receiving low-dose aspirin was significantly higher than in patients not receiving low-dose aspirin, with an odds ratio of 6.72 (95% confidence interval: 1.8-25.7) [19]. Regarding tumor size, Metz et al. reported that aspirin use was an independent risk factor of POH after EMR for colorectal lesions ≥ 20 mm in size, with an odds ratio of 6.3 [20]. Regarding anticoagulants, Hui et al. reported that warfarin use was an independent risk factor for EMR with an odds ratio of 13.37 (95% confidence interval: 4.10-43.65) [21]. In addition, we previously reported that anticoagulant use (warfarin and DOACs) was an independent risk factor of POH after endoscopic submucosal dissection (ESD) for colorectal neoplasia, with an odds ratio of 8.76 (95% confidence interval:1.24-30.19) [22]. Yamashita et al. also reported that the rate of POH after ESD for colorectal neoplasia in patients receiving anticoagulants (warfarin and DOACs) was higher than in patients not receiving such treatment [23]. Niikura et al. found in their analysis of the nationwide Japan Diagnosis Procedure Combination database that the rate of POH after therapeutic colonoscopy for colorectal neoplasia in patients receiving DOACs (rivaroxaban, edoxaban, and dabigatran) was significantly higher than that in patients not receiving DOACs. (15.3% (140/914) vs. 3.2% (11102/344632), *p* < 0.001) [8]. In contrast, Yu et al. reported that patients prescribed DOACs had no significantly increased risk of POH after EMR, with an odds ratio of 0.90 (95% confidence interval: 0.44–1.85) after adjusting for clinical background characteristics [24]. Conversely, Yanagisawa et al. reported that, in a retrospective study conducted at a single institution, the rate of POH after polypectomy for colorectal neoplasia in patients receiving DOACs (rivaroxaban, apixaban, edoxaban, and dabigatran) was significantly higher than that in patients not receiving any antithrombotics (13.8% (10/73) vs. 0.9% (2/218), *p* < 0.001) [25].

In the present study, we evaluated four types of DOACs and reported for the first time the high rate of POH about DOAC patients after EMR in a multicenter study.

Previous studies regarding the risk of non-procedural-related GI bleeding in DOAC users compared to warfarin have produced varied results, depending on the type of DOAC evaluated. For example, dabigatran at 150 mg twice daily, edoxaban at 60 mg once daily, and rivaroxaban increased non-procedural-related GI bleeding compared to warfarin [26–28]. In contrast, a study showed that apixaban did not increase POH [29]. In the current study, while the rate of POH in the DOAC group was higher than that in the warfarin group, this difference did not reach statistical significance probably due to poverty of case number.

Regarding risk factors for POH besides antithrombotic drugs, several reports have found that the tumor size was a risk factor [30–32]. In the present study, only patients with DOAC were analyzed, and a larger polyp size was found to be a risk factor of POH. Especially, tumor size for 3POH cases in the DOAC group was ≥20 mm. To our knowledge, this is the first report to address risk factors for POH in patients receiving DOACs.

Regarding the types of DOACs, no significant differences in the rate of POH after EMR per lesion were noted among DOACs, although none of the patients receiving edoxaban, the most recently developed DOAC, showed POH. Yanagisawa et al. reported that there was no significant difference in the rate of POH by type of DOAC, but the number of cases was rather small in their study [25]. Regarding dabigatran, it is a prodrug, unlike other DOACs, and remains as a prodrug in the GI tract, there is possibility that dabigatran may be transformed into its active form by intestinal bacteria and thus inhibits hemostasis [33]. Furthermore, dabigatran is the only anticoagulant with a higher ratio of lower GI bleeding than upper GI bleeding [34]. Dabigatran may therefore be associated with a high hemorrhage risk after EMR as well. Further studies are expected for proving the difference of POH about each DOACs.

Several limitations associated with the present study. This was a retrospective study. Some data such as the polyp size and locations in each group was examined only in cases with detail information from medical records. The materials used—such as the injection solution and snares—as well as the electrosurgical unit settings differed among institutions. The sample size was quite small, although the number of lesions in the DOAC group exceeded 100 cases. The mean age in the no antithrombotics group was significantly younger than that in the other antithrombotics group. This difference in the patient background may have affected the rate of POH. In Japan, lesions ≤ 10 mm in size were resected by either hot polypectomy or EMR, according to the endoscopist's preference. Recently, most cases have been resected by cold snare polypectomy. In the 8 institutions that participated in this study, endoscopists performed EMR for colorectal lesions < 10 mm in size in order to perform en bloc and R0 resection.

## 5. Conclusion

Patients receiving DOACs had higher rates of POH after EMR for colorectal lesions than those not receiving any antithrombotics. A larger tumor size especially tumor size ≥ 20 mm was a risk factor of POH in patients with DOACs. The number of DOAC patients is increasing as the elderly population grows. We must be careful when prescribing endoscopic therapy for these patients. The further accumulation of data is needed in order to determine whether or not to extend the discontinuation period of DOACs and to close EMR ulcers endoscopically to prevent POH in patients receiving DOACs.

## Figures and Tables

**Table 1:** Clinical characteristics of patients receiving endoscopic mucosal resection in the DOAC, warfarin, antiplatelet, and no antithrombotics groups.

	Total	DOAC group	Warfarin group	Antiplatelet group	No antithrombotics group
Case number	2062	63	34	185	1780
Age (years), mean ± SD (range, min-max)	67.2 ± 11.0 (20-93)	74.2 ± 7.0^∗^ (57-88)	73.2 ± 6.7^∗^ (52-86)	72.1 ± 8.8^∗^ (41-93)	66.3 ± 11.1 (20-93)
Sex: M/F (% (*n*))	63.2/36.8 (1304/758)	66.7/33.3 (42/21)	67.6/32.4 (23/11)	75.1/24.9^∗^ (139/46)	61.8/38.2 (1100/680)
Mean polyp size (mm), mean ± SD (range, min-max)	7.4 ± 4.6 (3-30)	7.5 ± 5.3 (3-30)	7.6 ± 4.3 (3-20)	7.4 ± 3.4 (3-18)	7.1 ± 3.9 (3-18)
Rate of right-sided colon (%)	51.8 (132/255)	48.1 (65/135)	41.2 (7/17)	48.1 (25/52)	68.6 (35/51)
Coprescription of antiplatelet (%(*n*))	N/A	17.5 (11)	23.5 (8)	N/A	N/A

^∗^vs. no antithrombotics group: *p* < 0.001. DOAC: direct oral anticoagulant; M: male; F: female; min: minimum; max: maximum; right-sided: cecum to transverse colon; N/A: not applicable.

**Table 2:** The comparison between cases with and without POH after endoscopic mucosal resection in patients with DOACs.

	DOAC users POH	DOAC users No POH	*p* value
Lesion number	5	130	
Morphology (% (*n*)) Polypoid/nonpolypoid	80.0 (4)/20.0 (1)	86.2 (112)/13.8 (18)	0.69
Tumor size (mm), mean (range, min-max)	16.2 ± 8.3 (4-25)	7.2 ± 4.9 (3-30)	<0.001
Rate of size ≥ 20 mm (% (*n*))	60.0 (3)	4.6 (6)	<0.001
Tumor location (% (*n*)) Right-sided/left-sided/rectum	60.0/20.0/20.0 (3/1/1)	48.5/42.3/9.2 (63/55/12)	0.52
Histology (% (*n*)) (SSAP/Ad/HGD/T1/others)	0/40.0/60.0/0/0 (0/2/3/0/0)	3.8/83.8/7.7/2.3/2.3 (5/109/10/3/3)	0.37
Coprescription of antiplatelet (% (*n*))	0 (0)	14.6 (19)	0.35

DOAC: direct oral anticoagulant; POH: postoperative hemorrhage; min: minimum; max: maximum; right-sided: cecum to transverse colon; left-sided: descending colon to sigmoid colon; SSAP: sessile serrated adenoma and polyp; Ad: low-grade adenoma; HGD: high-grade dysplasia.

**Table 3:** Details of DOAC patients with POH after endoscopic mucosal resection.

No.	Age (years)	Sex	Tumor location	Tumor size (mm)	Morphology (polypoid/nonpolypoid)	DOAC	Coprescription with antiplatelet	Histology	Number of POH	Hemorrhage date (day)	Nature of hemorrhage
1	75	M	T	20	Polypoid	Rivaroxaban	No	Ad	2	1 and 4	Apparent bleeding
2	68	M	R	20	Nonpolypoid	Apixaban	No	HGD	1	6	Apparent bleeding
3	76	M	T	25	Polypoid	Apixaban	No	HGD	2	2 and 3	Apparent bleeding
4	88	M	S	12	Nonpolypoid	Rivaroxaban	No	HGD	1	10	Apparent bleeding
5	70	M	A	4	Polypoid	Dabigatran	No	Ad	1	6	Apparent bleeding

DOAC: direct oral anticoagulant; POH: postoperative hemorrhage; M: male; F: female; T: transverse colon; R: rectum; S: sigmoid colon; A: ascending colon; Ad: low-grade adenoma; HGD: high-grade dysplasia.

## Data Availability

The patient data used to support the findings of this study are available from the corresponding author upon request.
